# Characterization and Comparative Analysis of Mitochondrial Genomes Among the Calliphoridae (Insecta: Diptera: Oestroidea) and Phylogenetic Implications

**DOI:** 10.3389/fgene.2022.799203

**Published:** 2022-02-17

**Authors:** Yanjie Shang, Lipin Ren, Xiangyan Zhang, Yi Li, Changquan Zhang, Yadong Guo

**Affiliations:** Department of Forensic Science, School of Basic Medical Sciences, Central South University, Changsha, China

**Keywords:** blowflies, Oestroidea, mitochondria, phylogeny, Calyptratae

## Abstract

The Calliphoridae (blowflies) are significant for forensic science, veterinary management, medical science, and economic issues. However, the phylogenetic relationships within this family are poorly understood and controversial, and the status of the Calliphoridae has been a crucial problem for understanding the evolutionary relationships of the Oestroidea these years. In the present study, seven mitochondrial genomes (mitogenomes), including six calliphorid species and one Polleniidae species, were sequenced and annotated. Then a comparative mitochondrial genomic analysis among the Calliphoridae is presented. Additionally, the phylogenetic relationship of the Calliphoridae within the larger context of the other Oestroidea was reconstructed based on the mitogenomic datasets using maximum likelihood (ML) and Bayesian methods (BI). The results suggest that the gene arrangement, codon usage, and base composition are conserved within the calliphorid species. The phylogenetic analysis based on the mitogenomic dataset recovered the Calliphoridae as monophyletic and inferred the following topology within Oestroidea: (Oestridae (Sarcophagidae (Calliphoridae + (Polleniidae + (Mesembrinellidae + Tachinidae))))). Although the number of exemplar species is limited, further studies are required. Within the Calliphoridae, the Chrysomyinae were recovered as sister taxon to Luciliinae + Calliphorinae. Our analyses indicated that mitogenomic data have the potential for illuminating the phylogenetic relationships in the Oestroidea as well as for the classification of the Calliphoridae.

## Introduction

The Oestroidea is a large and ecologically diverse clade within the order Diptera. Resolving the phylogenetic relationships of the Oestroidea is complicated, with little agreement on the monophyletic status based on morphology- and molecular-based studies (Pape and Arnaud., 2001; Winkler et al., 2015; Zhang et al., 2016; Marinho et al., 2017). The composition of the Oestroidea ranges from six families (Calliphoridae, Mystacinobiidae, Rhinophoridae, Tachinidae, Oestridae, and Sarcophagidae) (Marinho et al., 2012) to nine families (Calliphoridae, Mystacinobiidae, Mesembrinellidae, Oestridae, Rhinophoridae, Rhiniidae, Tachinidae, Sarcophagidae, and most recently, the Ulurumyiidae) (Cerretti et al., 2017; Michelsen and Pape, 2017).

Within the Oestroidea, the monophyly of the Sarcophagidae is well corroborated based on the morphology (Pape, 1996) and has been confirmed by molecular data (Shang et al., 2019; Ren et al., 2019). The monophyly of the family Oestridae is well supported based on the morphology (Pape, 2001), mitogenomic data (Zhang et al., 2016), and protein-encoding ultraconserved elements (UCEs) (Buenaventura et al., 2021). The Mesembrinellidae as a monophyletic is well corroborated based on morphological data (Toma and Carvalho, 1995) and five molecular markers (ITS2, 28S, COI, COII, and 16S) (Marinho et al., 2017). The monophyly of the Tachinidae is supported by the morphology (Pape, 1992) and four nuclear loci (7,800 bp) (Stireman et al., 2019). The Polleniidae are monophyletic based on 66 morphological traits and sequences from three nuclear protein-coding genes (CAD, MAC, and MCS) (Cerretti et al., 2019). However, there is still a large number of different phylogenetic hypotheses on the relationships between the families within the Oestroidea, some of them poorly supported, highlighting the need for further studies (Narayanan Kutty et al., 2019). Among its commonly recognized families, the controversial monophyletic status of the Calliphoridae is one of the key problems for understanding the phylogenetic relationships of the Oestroidea.

The Calliphoridae (known as blowflies) (Diptera: Calyptratae: Oestroidea) is a very heterogeneous and diverse group of medical, forensic, and veterinary importance, comprising approximately 97 genera and 1,500 species, of which many are distributed worldwide, while others show more localized distributions (Byrd and Castner, 2010; Zhang, 2013). The family is known for its saprophagous and myiasis-causing members in the Chrysomyinae, Calliphorinae, and Luciliinae (Stevens and Wallman, 2006).

Historically, the classification into tribes and subfamilies of the Calliphoridae has been contentious. The classification and composition of Calliphoridae subfamilies have gone through many variations (Kurahashi, 1989), ranging from five subfamilies (Calliphorinae, Chrysomyinae, Mesembrinellinae, Ameniinae, and Rhiniinae) (Hennig, 1973) to 13 subfamilies (Chrysomyinae, Calliphorinae, Luciliinae, Toxotarsinae, Melanomyinae, Auchmeromyinae, Bengaliinae, Polleniinae, Mesembrinellinae, Phumosiinae, Rhiniinae, Helicoboscinae, and Ameniinae) (Rognes, 1986; Rognes, 1991; Rognes, 1997). Yan et al. (2021) redefined the blowflies as the most inclusive monophylum within the superfamily Oestroidea not containing Mystacinobiidae, Mesembrinellidae, Polleniidae, Oestridae, Tachinidae, Sarcophagidae, and Ulurumyiidae, based on 2,221 single-copy nuclear protein-coding genes. The constituent subfamilies were reclassified as Bengaliinae, Ameniinae, Chrysomyinae, Calliphorinae, Phumosiinae, Luciliinae, Rhinophorinae stat. Rev., and Rhiniinae stat. rev. However, some researchers had previously proposed the subfamilies of the Calliphoridae, such as Rhiniidae, Mesembrinellinae, Bengaliinae, Polleniinae, and Rhinophoridae, to be elevated to family status (Lehrer, 2005; Kutty et al., 2010; Marinho et al., 2012; Cerretti et al., 2019), although there is still much controversy with these classifications and further studies are required. In addition, the monophyly and non-monophyly status of the Calliphoridae has also long been controversial. Some studies support the monophyletic status of the Calliphoridae (Yan et al., 2021), while others consider them non-monophyletic or paraphyletic (Marinho et al., 2012).

Mitochondrial genome (mitogenome) exhibiting the characteristics of small size, matrilineal inheritance, high copy numbers, a relatively high evolutionary rate, and protein-coding genes’ (PCGs’) sequence conservatism has become a powerful marker for phylogenetic relationships, evolutionary biology, specimen identification, and comparative genomics analysis (Aydemir and Korkmaz, 2020; Li et al., 2020). The mitogenome appears to evolve faster than the nuclear genome and is thought to be a reliable marker for a finer phylogenetic resolution (or higher branch support values) in fast-evolving groups (Cameron, 2014). Newly accessible mitochondrial genome data will be helpful for us to comprehend the characteristics of mitogenomes and the phylogenetic relationships of the Calliphoridae, even the Oestroidea. However, only 29 calliphorid species, including 56 mitogenome sequences, have been sequenced from the diversity of blowflies (GenBank, February 2021). The limited number of Calliphoridae mitogenome sequences is severely limiting our molecular analysis of the species at the genomic level.

Therefore, in this context, we determined seven mitogenomes, including six calliphorid species and one Polleniidae species (former Calliphoridae subfamily), and then provided the mitogenome characteristics and comparative mitogenome analysis of these species. We explored the identification power of mitogenomes for calliphorid species, and the molecular phylogenetic relationship of the Calliphoridae within the larger context of the other Oestroidea was first reconstructed based on the complete mitogenome datasets using different approaches for phylogenetic inference. This is important for the improvement of the databases of this significant calliphorid species, which strengthens our understanding of their phylogenetic relationships and contributes to taxonomic diagnosis.

## Materials and Methods

### Sample Collection and Identification

Adult specimens of *Lucilia papuensis*, *Polleniopsis mongolica*, *Triceratopyga calliphoroides*, *Calliphora sinensis*, *Calliphora uralensis*, *Calliphora nigribarbis* (Diptera: Calliphoridae), and *Pollenia pediculata* (Diptera: Polleniidae) were tempted and collected by pig liver carrion in Jining, Shandong Province, China (35°24′29.80″N, 116°34′30.96″E) in April and June 2019. These specimens were killed by freezing, and then identified through traditional morphological keys (Lu and Wu 2003). All test tube samples were assigned a unique label and kept in Guo’s Lab, Central South University (CSU).

### DNA Extraction and Sequencing

Total genomic DNA from each specimen preserved individually in 95–100% ethanol was extracted using the TIANamp Micro DNA Kit (TIANGEN BIOTECH CO., LTD.), following the manufacturer’s protocol. DNA content was quantitated by a NanoDrop 2000 (Thermo Scientific, United States). The DNA library of each specimen was constructed following the manufacturer’s protocol of the Illumina^®^ TruSeq^®^ DNA Sample Preparation Kit (Illumina, San Diego, United States), with an insert size of 250 base pairs (bp). Paired-end sequencing with a read length of 150 bp (PE 150 bp) was performed on an Illumina Hiseq 2,500 platform at OE Biotech. Co., Ltd (Shanghai, China).

### Sequence Assembly, Annotations, and Analysis

Each sequenced sample generated at least 2 Gb of raw reads, and the mean length of reads was about 125 bp. The quality of raw reads was checked with the software fastp v0.23.2 to ensure the reliability of the obtained data (Chen et al., 2018), and then trimmed and filtered using Trimmomatic v0.36 (Bolger et al., 2014). Reads shorter than 88 bp and ambiguous bases (N) were removed. The sequencing quality was controlled using the NGSQC-Toolkit v2.3.3 software (avg. Q30 > 80%, avg. Q20 > 85%) (Patel and Jain, 2012). The MitoZ software v2.3 https://github.com/linzhi2013/MitoZ, with modified SOAPdenovo-Trans algorithms and Quick mode settings, was used for the mitochondrial *de novo* assembly using the high-quality filtered reads (Xie et al., 2014; Meng et al., 2019).

**FIGURE 1:**
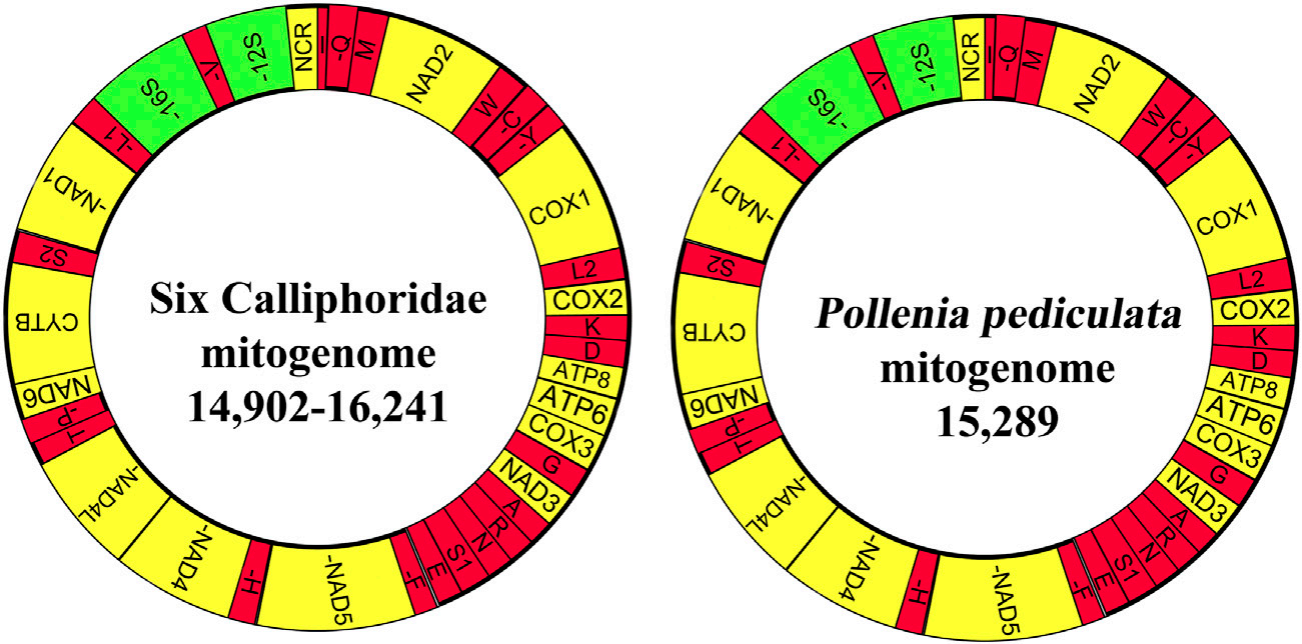
Graphical map of the mitogenome of seven mitogenomes, including six calliphorid species and one Polleniidae species. Protein coding and ribosomal RNA genes are shown using standard abbreviations. tRNAs are abbreviated *via* a single letter, covering L1 = CUN, L2 = UUR, S1 = AGN, S2 = UCN, and NCR = putative control region. The 13 PCGs and control region are yellow, tRNAs are red, and rRNAs are green. Encoding genes in the N-strand are marked with dashes (−).

The mitogenomic boundaries and 13 PCGs were checked by alignment with previously published mitogenomes of flies using Muscle (codons) in MEGA X v 11.0.10 (Kumar et al., 2018). The open reading frames (ORFs) were predicted with the NCBI ORFfinder using the invertebrate mitochondrial genetic code. The MITOS Web Server (Lambert et al., 2004) and tRNAscan-SE Search Server v1.3.1 (Lowe and Chan, 2016) were used to predict and identify rRNAs and tRNAs. The long non-coding control region (putative A + T-rich region) was identified online by the Tandem Repeats Finder v 4.10.0 (Benson, 1999). Mitogenome maps were produced using OGDRAW v1.3.1 (Greiner et al., 2019) (Figure 1). The seven newly sequenced mitogenomes were submitted to GenBank (accession numbers: MT017724, MT017722, MT017707, MT017731, MT017721, MT017717, and MT017729).

The nucleotide base composition and relative synonymous codon usage (RSCU) values were calculated with MEGA X v 11.0.10 (Kumar et al., 2018). Strand asymmetry was calculated by the AT Skew and GC Skew as outlined by previous studies (Shang et al., 2019). Genetic distances among calliphorid species were calculated in MEGA X by the pairwise p-distance model. The mean of non-synonymous nucleotide substitutions per non-synonymous site (Ka), synonymous nucleotide substitutions per synonymous site (Ks), and evolutionary rate (Ka/Ks, *ω*) of 13 PCGs were calculated by DnaSP v6.12.03 (Rozas et al., 2017). Nucleotide saturation was evaluated by plotting the transitions (Ti) and transversions (Tv) with a GTR model in DAMBE v6.4.79 (Xie et al., 2014) (Supplementary Figure S1). The sequence divergence heterogeneity within datasets was evaluated using AliGROOVE v.107 (Kück et al., 2014) with the default setting for phylogenetic tree building (Liu et al., 2018) (Supplementary Figure S2).

### Phylogenetic Analyses

The phylogenetic analyses were conducted using the sequences of 13 PCGs (excluding the termination codons) and two rRNAs of 63 calliphorid specimens (34 species), and an additional 18 species of Oestroidea as well as five Muscidae species (Muscoidea) serving as the outgroup (Supplementary Table S1) (Fu et al., 2016; Karagozlu et al., 2019; Ramakodi et al., 2015; Ren et al., 2016; Rodrigue and Lartillot, 2014; Seo et al., 2019; Shao et al., 2012; Shi et al., 2016; Stevens et al., 2008; Yan et al., 2016a; Yan et al., 2016b; Yan et al., 2019; Zhu et al., 2016). The genes of 13 PCGs and two rRNAs were aligned using MAFFT online v7.471 (Katoh et al., 2019). Three datasets were concatenated by SequenceMatrix to further reconstruct phylogenetic analysis (Vaidya et al., 2011): 1) PCG12 (first and second codon positions of PCGs), 2) PCG123 (all codon positions of PCGs), and 3) PCG123rRNA (all codon positions of 13 PCGs combined with two rRNAs).

The ML (maximum-likelihood) analysis was executed using IQ-TREE v1.6.2 (Nguyen et al., 2015). Optimal partitioning scheme for each dataset and the best evolutionary model for each partition were selected according to the Bayesian information criterion (BICc) (Supplementary Table S2), and the branch support values of majority-rule consensus tree were inferred with 10,000 bootstrapped replicates (BPs). The results showed that the best models of subset partitions were almost identical, which could be merged into the dataset. The best model of GTR + I + G was chosen. For BI analyses, the optimal partitioning scheme for each dataset and the best model for each partition were determined using PartitionFinder v2.0 with the corrected Akaike information criterion (AICc) and the “greedy” algorithm with branch lengths estimated as “unlinked” to search for the best-fit scheme. The GTR + I + G model was the best-fit model for nucleotide alignments (Lanfear et al., 2017) (Supplementary Table S3). The BI (Bayesian inference) analyses were performed with MrBayes v3.2.4 (Ronquist et al., 2012), with the following conditions following the instructions given by Ren et al. (2019): sampling every 1,000 generations in 100 million generations and discarding the first 25% as burn-in. The Tracer v. 1.7 software (Rambaut et al., 2018) was used to examine the sufficient parameter sampling [estimated sample size (ESS) > 200], and posterior probabilities (PP) were used to assess the branch support values of the BI tree. The ML and BI phylogenetic trees were visualized using FigTree v.1.3.1 (Rambaut, 2020).

## Results and Discussion

### Mitogenomic Structure and Composition

The newly obtained mitogenomes of six calliphorid and one polleniid species were from 14,902 bp (*T. calliphoroides*) to 16,241 bp (*C. nigribarbis*) and 15,289 bp (*P. pediculata*) in length (Supplementary Table S4), and formed the typical circular double-stranded DNA molecule containing 37 genes (2 rRNA genes, 13 PCGs, and 22 tRNA genes) and an A + T-rich region; 23 genes were located on the majority strand (J-strand) (9 PCGs and 14 tRNAs), while the remaining genes (4 PCGs, 8 tRNAs, and 2 rRNAs) were found on the minority strand (N-strand), which is the classical structure of the ancestral insect mitogenome order (Shang et al., 2019).

**FIGURE 2:**
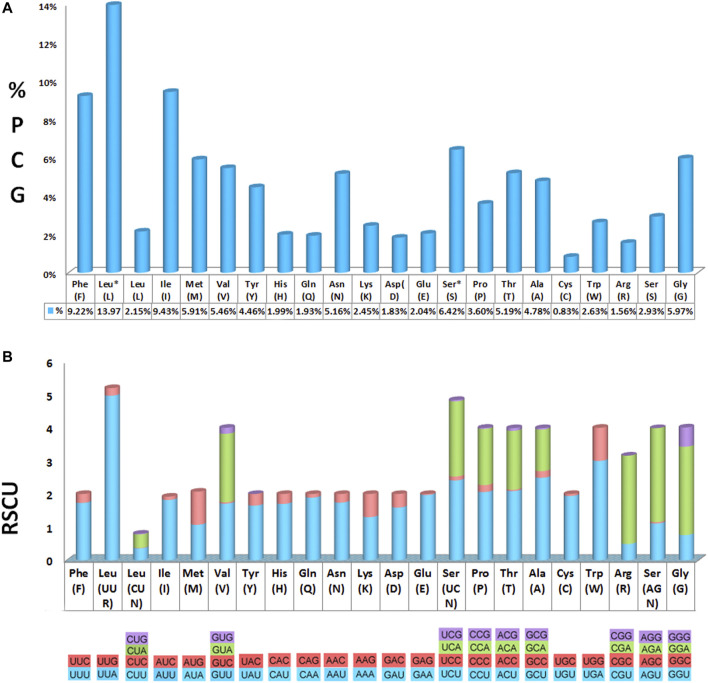
Amino acid distribution and relative synonymous codon usage (RSCU) in the *Calliphora nigribarbis* mitogenome. **(A)** Codon distribution. **(B)** RSCU. Codon families are provided on the *X* axis and the RSCU on the *Y* axis. This mitogenome presents all possible codon families existing in Diptera.

All 13 PCGs ranged from 164 bp (ATP8) to 1719 bp (ND5) in size and comprised 11,145 bp in total with stop codons removed, encoding 3,715 amino acids. For more details on initiation and termination codons, refer to Supplementary Table S5 and Supplementary Table S6 for details on the count and percentage of all codons used. Among the 22 amino acids, leucine (Leu), isoleucine (Ile), and phenylalanine (Phe) were the most frequently found, while cysteine (Cys) was the least frequent. Determining the RSCU revealed that the codons UUA, AUU, UUU, AAU, and AAA were the most frequently used, whereas the codons CCG, UCG, ACG, GCG, and CGC were rarely used (Figure 2, Supplementary Table S6).

**FIGURE 3:**
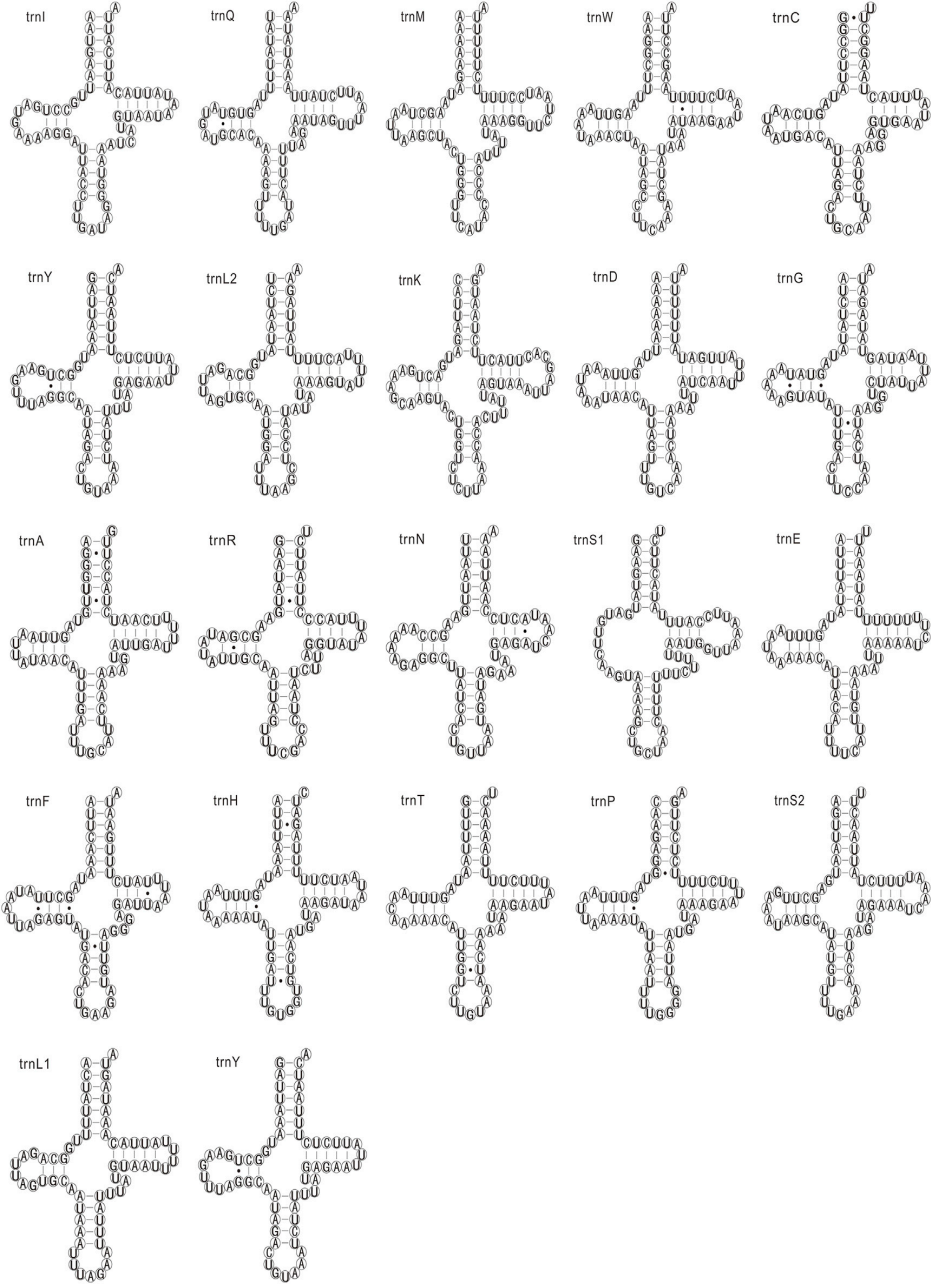
Inferred secondary structure of transfer RNAs (tRNAs) in the *Calliphora nigribarbis* mitogenome. All tRNA genes are shown in the order of occurrence in the mitochondrial genome starting from trnI. tRNAs are labeled with the abbreviations. Dashes (−) indicate Watson–Crick base pairing.

The 22 tRNAs in the seven newly sequenced mitogenomes were identified, which are typically discovered in most arthropod mitogenomes (Shang et al., 2019). The 21 tRNAs were folded into the typical clover-leaf structure, whereas the trnS1 had a special clover-leaf structure without a dihydrouridine (DHU) arm, which was replaced by a simple loop. The count and percentage of codon, and the codon usage of 13 PCGs of these mitogenomes are shown in Supplementary Table S6. The codon usage and predicted secondary structures of tRNAs of *C. nigribarbis* are shown in Figures 2, 3.

The large ribosomal subunit (16S rRNA) is located between tRNA-Leu (CUN) and tRNA-Val, whereas the small (ribosomal) subunit (12S rRNA) is located between tRNA-Val and the A + T-rich region. The A + T-rich region (also called long non-coding control region) is located between 12S rRNA and tRNA-Ile, and the control regions of these mitogenomes are greatly variable, varying from 88 bp (*T. a calliphoroides*) to 1427bp (*C. nigribarbis*) in length.

Interspecific distances and intraspecific divergence based on the data of COI, ND5, CYTB, 16S rRNA, 12S rRNA, and the combined sequences of the 13 PCGS and 2 rRNAs were calculated for sequence comparison, and the pairwise distance matrix of 34 calliphorid species is shown in Supplementary Table S7. The interspecific divergences between many calliphorid species were higher than 5.0% based on COI genes, which is consistent with previous reports (Sharma et al., 2015), and further illustrated that the application of COI segments for species identification can be achieved for most common calliphorid species. The sister species *L. illustris* and *L. caesar* are well known for their difficult identification, and showed the interspecific distance (COI:1.7%, ND5:1.7%, CYTB:1%, 12S rRNA:0.2%, 16S rRNA:0.0%,13PCGs+2rRNAs:1.4%) in our study, which is consistent with the result of 1.4–1.9% from COI sequences (Park et al., 2009) and 0.0–1.5% from COI barcode (GilArriortua et al., 2015). Therefore, mitochondria would not be a suitable molecular marker for the discrimination of the sister species.

### Nucleotide Composition, Heterogeneity, and Evolution Rates

The nucleotide composition showed strong bias toward A and T in the seven newly sequenced mitogenomes, with its content ranging from 76.02% (*T. calliphoroides*) to 79.84% (*P. mongolica*) (Supplementary Table S8). The analysis of average base composition at each codon position showed that the bias in AT content is much higher in the third codon position than in the first and second, an observation previously reported for other diptera groups, such as Sarcophagidae (Ren et al., 2020), Muscidae (Ren et al., 2019), and Oestridae (Zhang et al., 2016). The nucleotide compositional skew statistics indicated that the whole mitogenome had positive A-skew with negative G-skew in the seven newly sequenced mitogenomes. The AT-skew ranged from 0.01 to 0.03 and the GC-skew ranged from −0.19 to −0.15 in the whole mitogenome.

**FIGURE 4:**
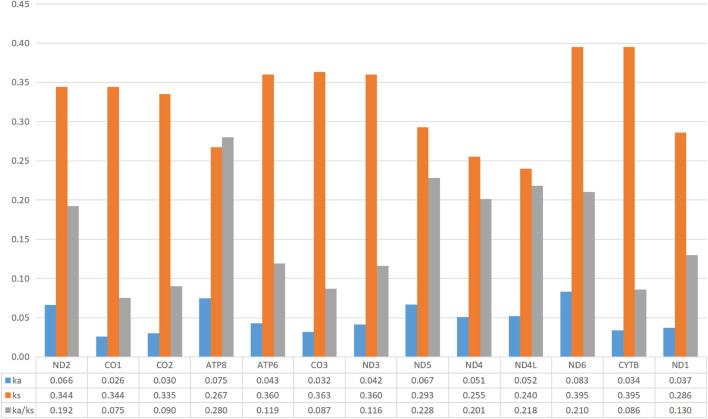
Evolutionary ratio of 13 PCGs, 2 rRNAs, and 22 tRNAs in the mitogenome of 35 calliphorid species. Synonymous nucleotide substitutions per synonymous site (Ks), non-synonymous nucleotide substitutions per non-synonymous site (Ka), and the ratio of Ka/Ks were calculated.

The heterogeneity of sequence divergence of various concatenated datasets was revealed with pairwise comparisons by AliGROOVE analyses based on the datasets PCG12, PCG123, and PCG12+2rRNAs, which resulted in low heterogeneity (Supplementary Figure S2). Moreover, to investigate the evolutionary pressure among 13 PCGs of 34 calliphorid species, the values of Ka, Ks, and ratio of Ka/Ks (*ω*) were calculated (Figure 4), with Ka < Ks, Ka = Ks, and Ka > Ks indicating purifying, neutral, and positive selection, respectively (Lynch and Conery, 2000). The average substitution rates in all mitogenome fragments of Ka varied from 0.026 (co1) to 0.083 (nd6), Ks varied from 0.240 (nd4l) to 0.395 (nd6, cytb), and Ka/Ks (*ω*) varied from 0.075 (co1) to 0.280 (atp8). The evolutionary rate *ω* values for 13 PCGs were less than 1.00, indicating that these genes are evolving under negative selection pressure in these calliphorid species. The COI gene exhibited the lowest evolutionary rate (*ω* = 0.075) of all the 13 PCGs, and can be assumed to be under strong negative selection.

### Phylogenetic Analysis

**FIGURE 5:**
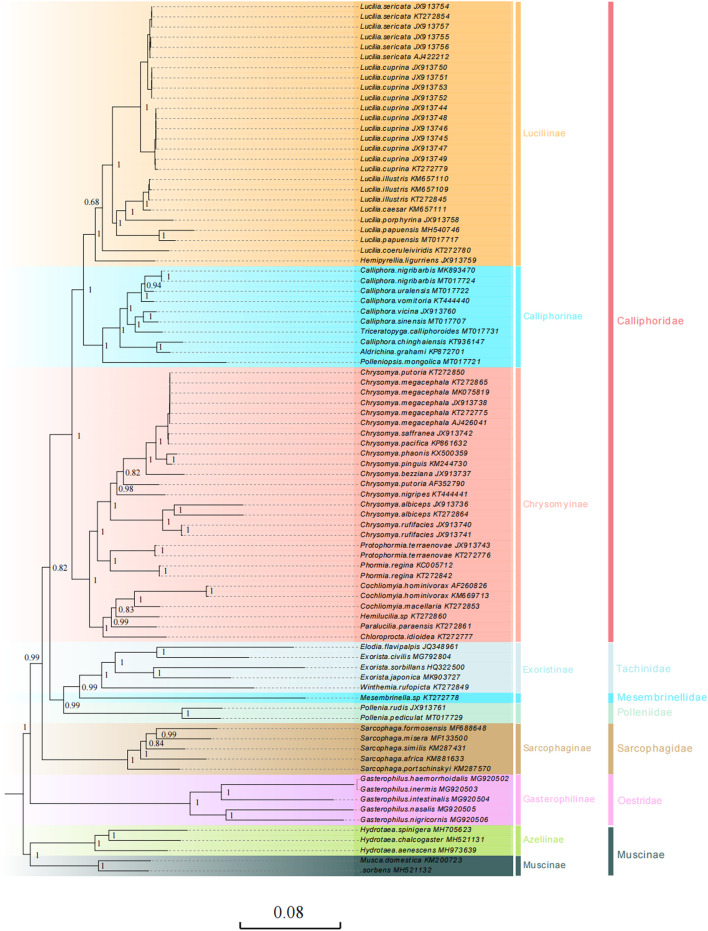
Phylogenetic analyses of 34 calliphorid species (including 63 mitogenomic sequences) within the larger context of the other 18 Oestroidea species were constructed based on the sequences of 13 PCGs (excluding the termination codons) and two rRNAs using Bayesian methods (BI). The five Muscidae species (Insecta: Diptera: Muscoidea) served as the outgroup. Numbers on branches are Bayesian posterior probabilities (PP). Different colors represent different subfamilies the species belong to. The two column taxonomic groups on the right side represent subfamilies and family according to the traditional morphological classification, respectively.

Here, we present the phylogenetic relationships of 34 calliphorid species (including 63 mitogenomic sequences) within the larger context of the other 18 Oestroidea species based on the three datasets: 1) PCG12, 2) PCG123, and 3) PCG123rRNA using the BI and ML methods (Figure 5, Supplementary Figures S3–S7). The five Muscidae species were the outgroup taxon of phylogenetic analysis. Saturation analyses showed that these datasets had no significant substitution saturation (Supplementary Figure S1).

The topologies resulting from the BI and ML analyses were highly consistent (Figure 5, Supplementary Figures S3–S7), with the phylogenetic relationships of Oestroidea clades generally recovered as (Oestridae + (Sarcophagidae + (Calliphoridae + (Polleniidae + (Mesembrinellidae + Tachinidae))))), which are slightly inconsistent with previous results; based on the ITS2, 28S, COI, and 16S regions, there are always two main clades in Oestroidea: [(Tachinidae + Mesembrinellinae) and (Rhiniinae, (Sarcophagidae + Calliphoridae)] (Marinho et al., 2012). The position of the Polleniidae and Mesembrinellidae in the Oestroidea is contentious. Historically, these groups have been treated as a subfamily of Calliphoridae (Pape, 1992; Rognes, 1997), but some authors have proposed that the Polleniidae and Mesembrinellidae groups have a monophyletic lineage separated from the Calliphoridae, giving family status to the group (Guimarães, 1977; Cerretti et al., 2019). This proposition was supported more recently by phylogenetic analyses based on molecular data (Kutty et al., 2010; Marinho et al., 2012; Singh and Wells, 2013; Yan et al., 2021). Our molecular phylogenetic relationship research based on mitogenomes also supports the family status of Mesembrinellidae and Polleniidae.

In our study, the sister relationships of Polleniidae and (Mesembrinellidae + Tachinidae) were supported (PP = 0.99; BP = 100) based on the most mitogenomic datasets, except for (Mesembrinellidae (Polleniidae, Tachinidae)) based on PCG12 using ML analyses (Supplementary Figure S5). Polleniidae is supported as the sister group to the family Tachinidae in most molecular-based phylogenetic analyses, based on COI, CAD, EF1*α*, 28 S rRNA (Singh & Wells 2013), UCEs (Buenaventura et al., 2021), nucleotide, and amino acid data for 1,456 single-copy protein-coding genes (Kutty et al., 2010), four nuclear loci (7,800 bp) (Stireman et al., 2019), and transcriptome and genomic data (Yan et al., 2021). In addition, the close relationship between Tachinidae and Mesembrinellidae has been supported using molecular analyses (Kutty et al., 2010).

The topology relationships of Oestridae have undergone differentiation based on molecule and morphology datasets, the Oestridae group is sister to the remaining Oestroidea, and sister to Tachinidae + Rhinophoridae (Pape, 1992). Our study supported for the group sister to remaining Oestroidea based on the mitogenomic datasets. The sister relationship between Sarcophagidae and Calliphoridae, although not widely corroborated, has been previously proposed by McAlpine (1989a), Ren et al. (2020). Most previous studies suggested a closer relationship between the Sarcophagidae and Tachinidae (Pape, 1992; Rognes, 1997; Tachi and Shima, 2010) or its placement with Mystacinobiidae, as a sister group to the remaining Oestroidea (Kutty et al., 2010). Our study supports a sister relationship between Sarcophagidae and Calliphoridae (PP = 0.99; BP = 100).

The Calliphoridae are a key lineage for reaching an understanding of the evolution and phylogeny of the Oestroidea (McAlpine, 1989b). For the Calliphoridae, most commonly proposed relationships place this family, albeit with different compositions, closer to the Rhinophoridae and Oestridae based on the morphology (Tschorsnig, 1985; Pape, 1992; Rognes, 1997) or as a sister taxon of the remaining Oestroidea based on the structure of the male postabdomen (Griffiths, 1972). Our study supports Calliphoridae as a sister to (Polleniidae (Mesembrinellidae, Tachinidae)) (PP = 0.99; BP = 100) based on the mitogenomic datasets. In addition, in our study, the Calliphoridae, being monophyletic, was supported based on PCG123 and PCG123rRNA (PP = 0.99; BP = 100) (Figure 5, Supplementary Figures S4,6,7), which is consistent with previous studies based on putative synapomorphies (McAlpine, 1989a), Pape, 1992), and single-copy nuclear protein-coding genes (Yan et al., 2021). The non-monophyly status of Calliphoridae is supported here based on PCG12 using BI and ML analyses (Supplementary Figures S3,S5). In addition, the non-monophyly status of Calliphoridae is supported based on morphological and molecular characteristics (Rognes, 1997; Kutty et al., 2010; Buenaventura et al., 2021), paraphyletic status is supported based on molecular phylogenetics (Marinho et al., 2012), and polyphyletic status is supported based on the mitochondrial and nuclear genes (Nelson et al., 2012; Singh and Wells, 2013).

Within the Calliphoridae, the sister-grouping relationship between Luciliinae and Calliphorinae was well supported (PP = 1.00; BP = 100), which corroborated previous studies (Kutty et al., 2010), and the monophyletic Luciliinae is in agreement with previously proposed hypotheses (Wallman et al., 2005; McDonagh 2009; Kutty et al., 2010; Marinho et al., 2012). The monophyly status of Calliphorinae was supported based on PCG123 and PCG123rRNA (PP = 0.99; BP = 100) (Figure 5, Supplementary Figure S4,6,7), which is consistently found in molecular systematic studies (Harvey et al., 2008; McDonagh and Stevens, 2011). The monophyly of the Chrysomyinae was well supported based on most mitogenomic datasets (PP = 1; BP = 100) (Figure 5, Supplementary Figures S4–8), which corroborated with that recently shown by Singh and Wells (2013), and Yan et al. (2021). but the non-monophyly status of Chrysomyinae based on PCG12 (Supplementary Figure S3) in our study and the study carried out by Kutty et al. (2010) found it to be para- or polyphyletic. At the genus level, the monophyletic status of *Lucilia* genus was strongly supported based on PCG123rRNA by BI and ML analyses, PCG123 by ML analyses (PP = 1; BP = 100) (Figure 5, Supplementary Figure S6,7), and non-monophyly status based on PCG12 (Supplementary Figures S3–5). The paraphyletic *Calliphora* genus was recovered based on these mitogenomic datasets. In addition, we corroborate previous findings (Junqueira et al., 2016) that the mitogenomic data KT272850, which is stated to be *C. Putoria*, after the carefully carried out sequence alignment and verification, is identical to *C. megacephala*.

The Calliphoridae phylogenies based on datasets with and without tRNA genes are almost identical, except for slight differences in some branch supports and branch lengths, irrespective of the analytic methods, which is similar to the study by Zhang et al. (2016).

The results of phylogenetic analyses based on the PCG123 or PCG123rRNA of these calliphorid species were similar to the traditional morphological classification and recent molecular studies (Harvey et al., 2008; Yan et al., 2021), which suggested that mitochondria were candidate molecular markers for analyzing phylogenetic relationships of Calliphoridae, even for Oestroidea species. However, in the present study, we found that these topological differences are mainly due to excluding the third codon positions of PCGs (PCG12) and the *Polleniopsis mongolica* (Calliphoridae) clustered together with Tachinidae. The Calliphoridae families and Calliphorinae subfamilies had non-monophyly status based on PCG12 using BI and ML analyses (Supplementary Figures S3,S5). The third codon positions are always excluded when constructing phylogenetic analysis. However, in the present study, the nucleotide substitution saturation of PCGs indicated that the third codon position was not saturated, or that there was partial saturation for transversions in most cases (Supplementary Figure S1). Caravas and Friedrich, 2013 estimated the performance of the third codon position in Diptera, showing that the third codon can still resolve some recent clades within the Calyptratae. As mentioned previously, the third codon positions of PCGs are important values in phylogenetic reconstruction within Oestroidea and should not be trimmed.

Moreover, because some former calliphorid subfamilies have been given family status, blowflies have undergone many changes in the number and composition of subfamilies (Kurahashi, 1989; Yan et al., 2021). Only a small part of the diversity in the Calliphoridae is involved in this study. The mitogenomic data of other Calliphoridae subfamilies are still lacking; therefore, further works that increase the number of other Calliphoridae subfamily mitogenomes for the monophyletic status and phylogenetic relationships of Calliphoridae are suggested.

## Conclusion

Mitogenomes provide informative molecular markers for phylogenetic analysis in the Calliphoridae. When adding the conserved rRNA genes, the topology is hardly changed, except for slight changes in node supports, and the third codon positions of PCGs are not saturated; these are important values for phylogenetic reconstruction within the Oestroidea. In order to make the phylogenetic relationship more stable and further improve the phylogenetic resolution, more taxa of the Calliphoridae should be added in the future.

## Data Availability

The datasets presented in this study can be found in online repositories. The names of the repository/repositories and accession number(s) can be found below: https://www.ncbi.nlm.nih.gov/genbank/, MT017724, MT017722, MT017707, MT017731, MT017721, MT017717, and MT017729.

## Abbreviations

ATP6 and ATP8, ATPase subunits 6 and 8; COI–COII, cytochrome *c* oxidase subunits I–II; Cytb, apocytochrome b; ND1–ND6 and ND4L, NDH dehydrogenase subunits one to six and 4L; rRNA, ribosomal RNA; PCGs, protein-coding genes; tRNA, transfer RNA.
