# Supplementary material for: Characterization and Comparative Analysis of Mitochondrial Genomes Among the Calliphoridae (Insecta: Diptera: Oestroidea) and Phylogenetic Implications
Source: Front Genet. 2022 Feb 17;13:799203. doi: 10.3389/fgene.2022.799203 (PMC8891575; doi:10.3389/fgene.2022.799203)
Supplement: Supplementary file 2 [file DataSheet1.PDF]

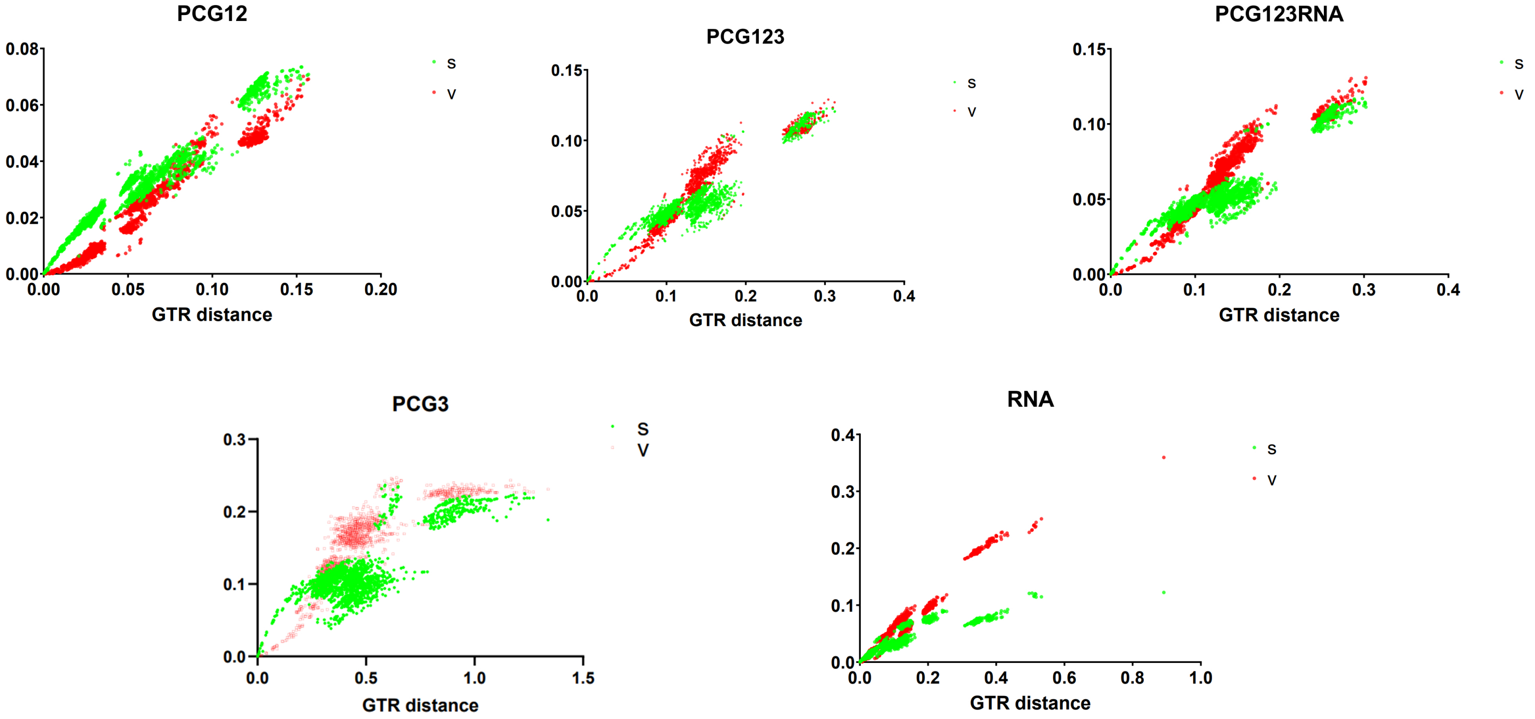

Fig.S1. Saturation analyses based the 13PCGs and 2 rRNAs in the Oestroidea mitogenomes (including 34 calliphoridae species and other 18 Oestroidea species). The x-axis represents pairwise distance estimated by GTR model, the y-axis is the absolute number of transitions and transversions. Transitions are shown as red circles and transversions as green circles.

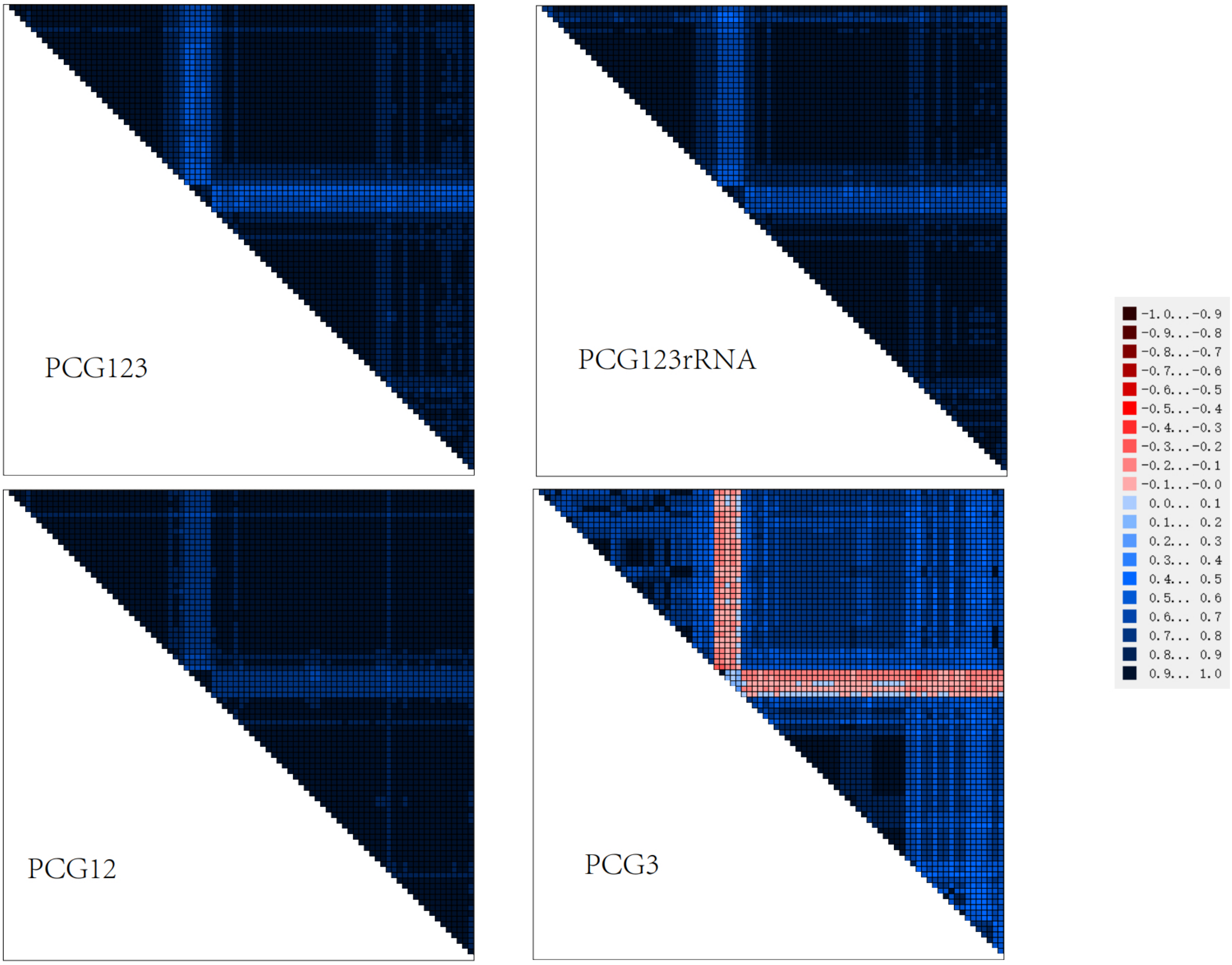

Fig.S2. Heterogeneous sequence divergence within datasets of Oestroidea mitogenomes (including 35 calliphoridae species and other 18 Oestroidea species). The obtained mean similarity score between sequences was represented by a colored square.

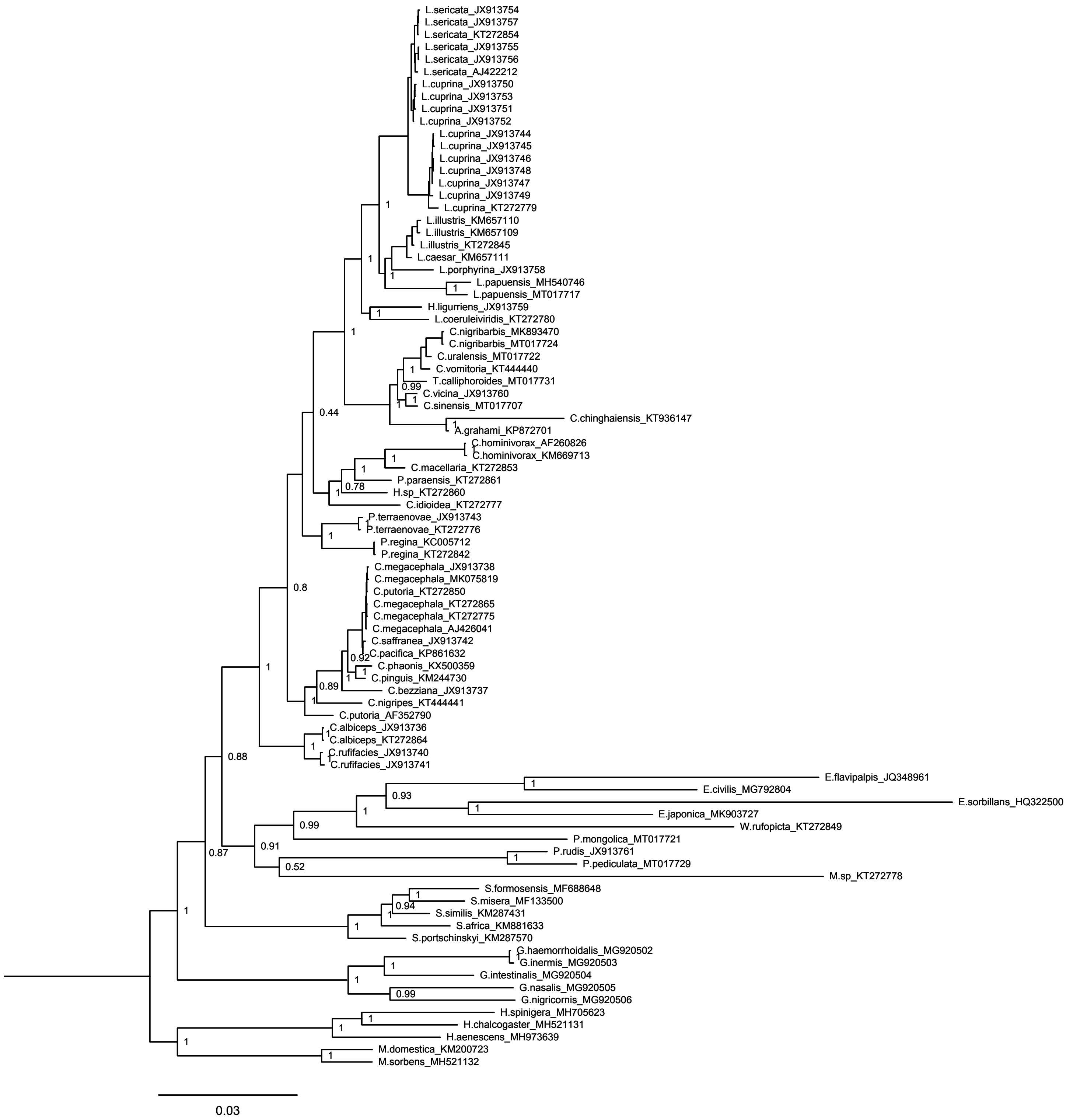

Fig.S3. The Phylogenetic analyses of 34 calliphoridae species (including 63 mitogenomes sequences) within the larger context of the other 18 Oestroidea species were constructed based on the sequences of PCG12 (first and second codon positions of PCGs) using Bayesian methods (BI). Numbers on branches are Bayesian posterior probabilities (PP).

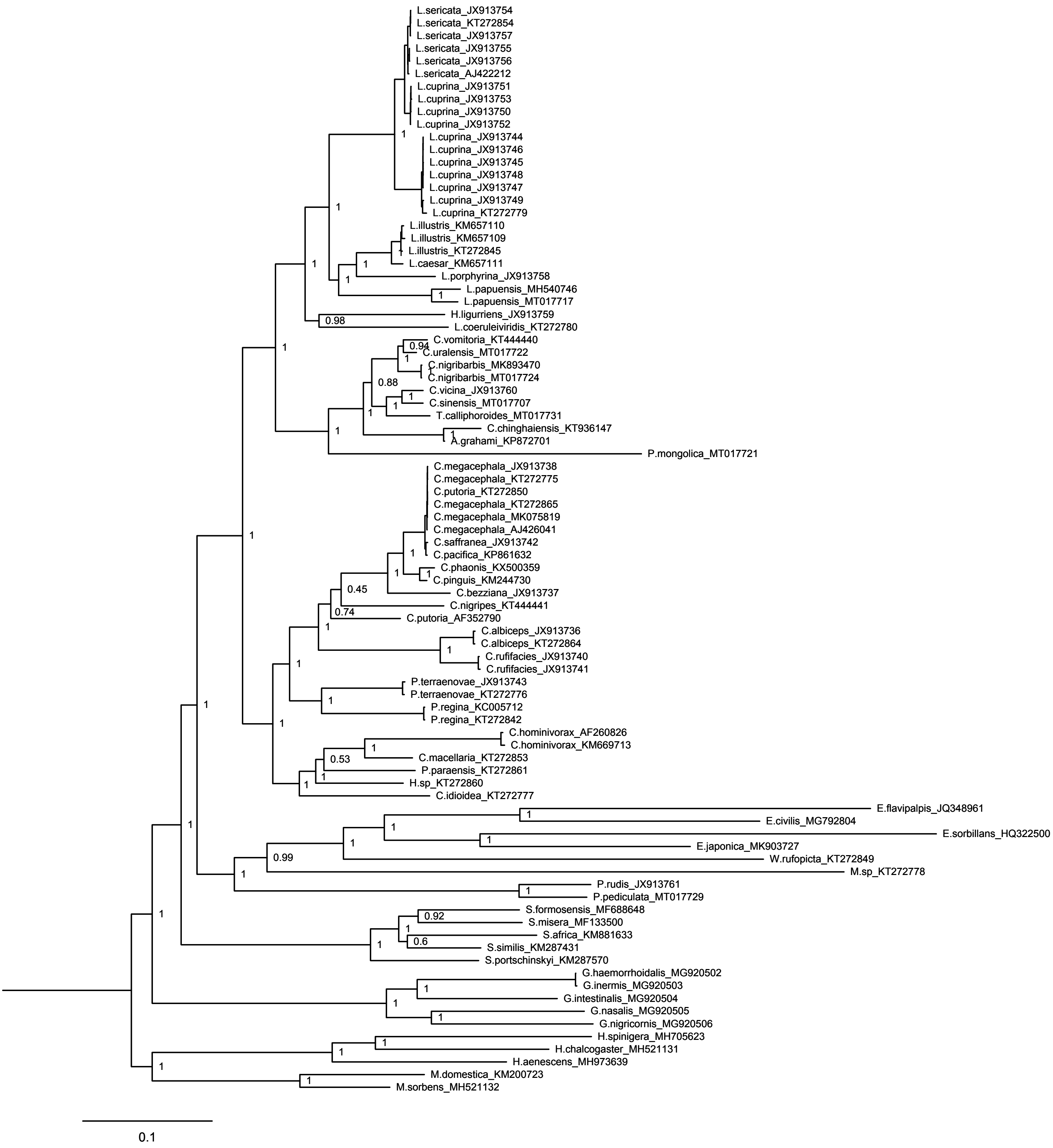

Fig.S4. The Phylogenetic analyses of 34 calliphoridae species (including 63 mitogenomes sequences) within the larger context of the other 18 Oestroidea species were constructed based on the sequences of PCG123 (all codon positions of PCGs) using Bayesian methods (BI). Numbers on branches are Bayesian posterior probabilities (PP).

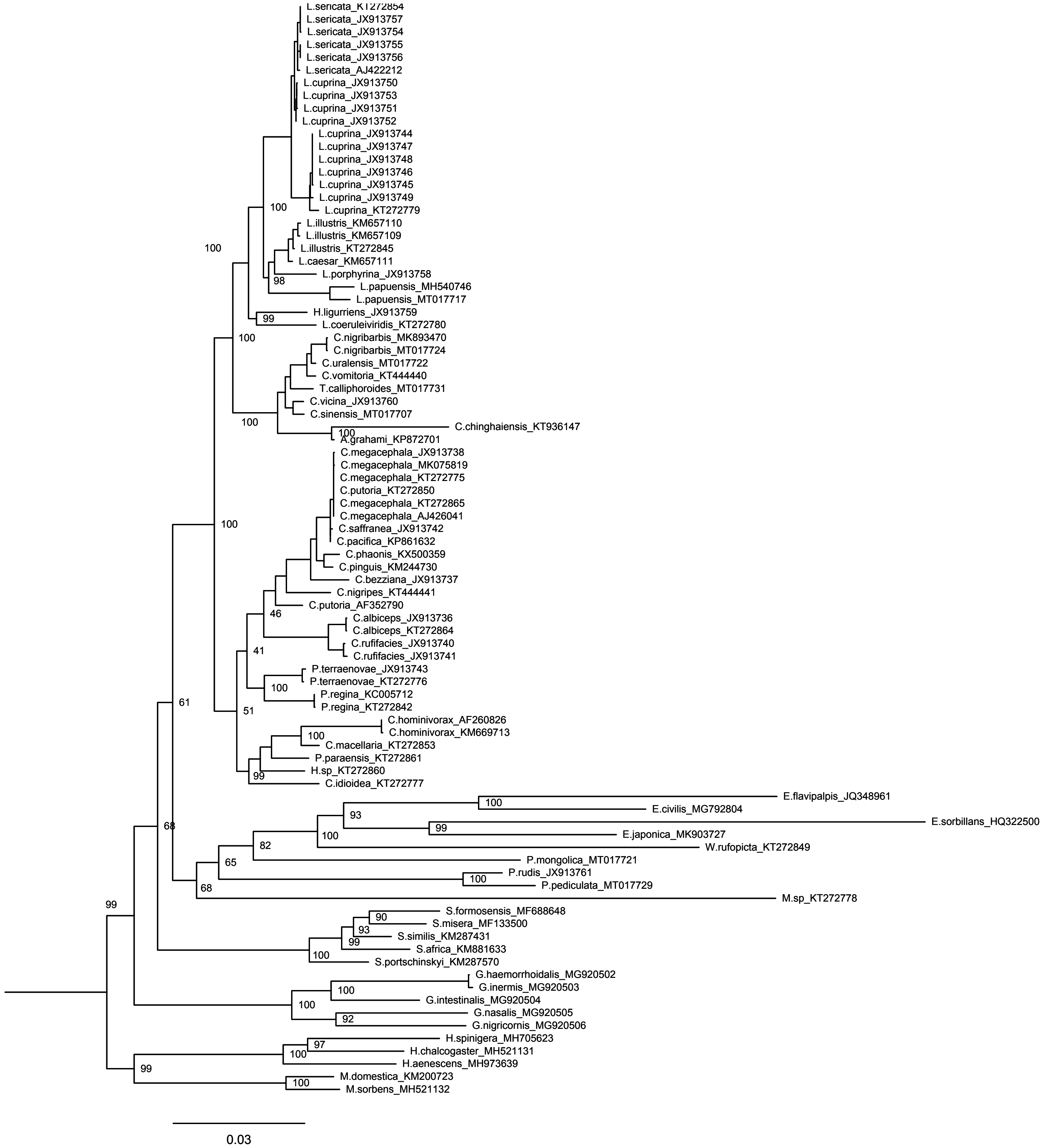

Fig.S5 The Phylogenetic analyses of 34 calliphoridae species (including 63 mitogenomes sequences) within the larger context of the other 18 Oestroidea species were constructed based on the sequences of PCG12 (first and second codon positions of PCGs) using maximum likelihood (ML) methods. Numbers on branches are bootstrapping replicates (BP).

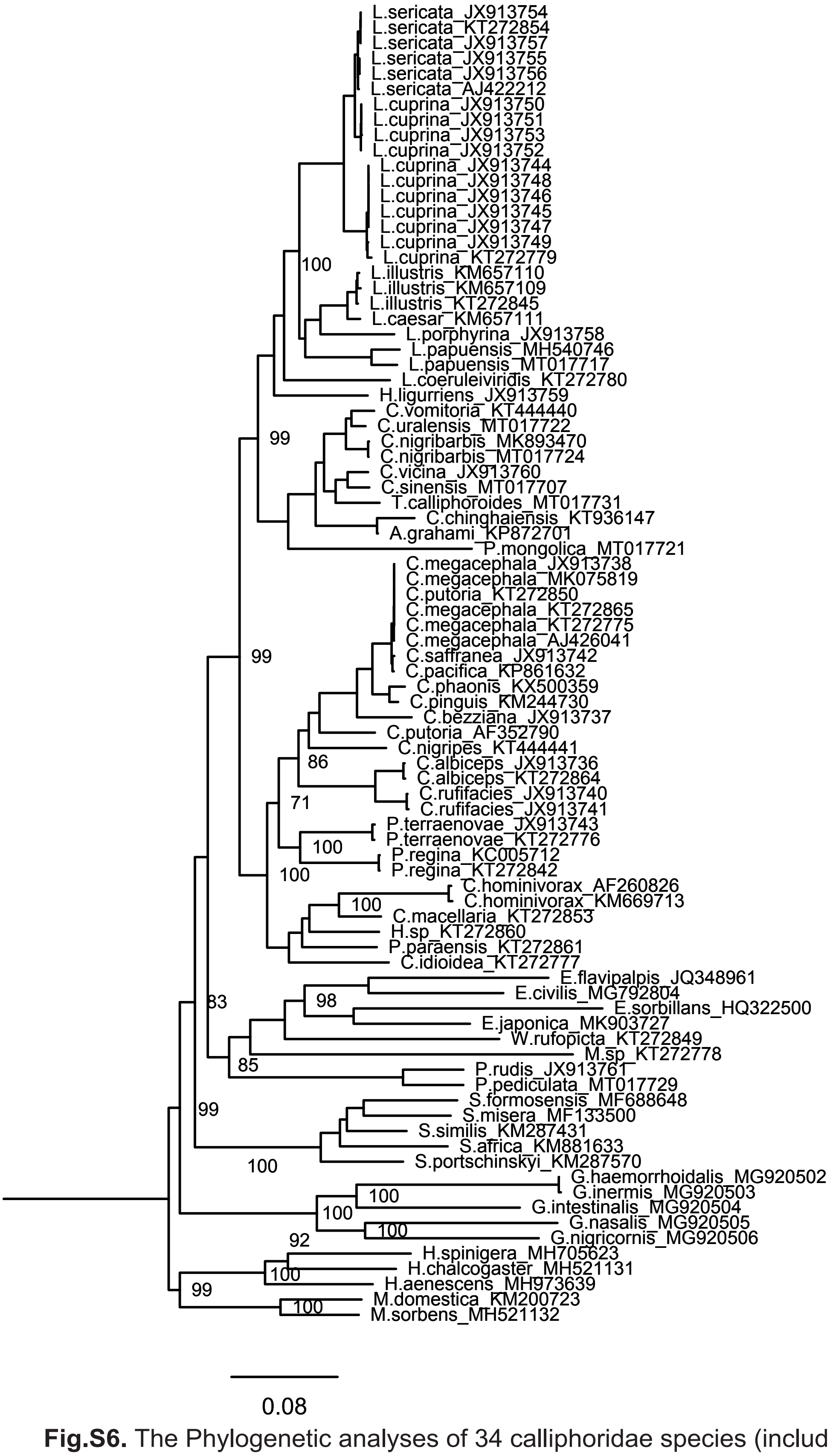

Fig.S6. The Phylogenetic analyses of 34 calliphoridae species (including 63 mitogenomes sequences) within the larger context of the other 18 Oestroidea species were constructed based on the sequences of PCG123 (all codon positions of PCGs) using maximum likelihood (ML) methods. Numbers on branches are bootstrapping replicates (BP).

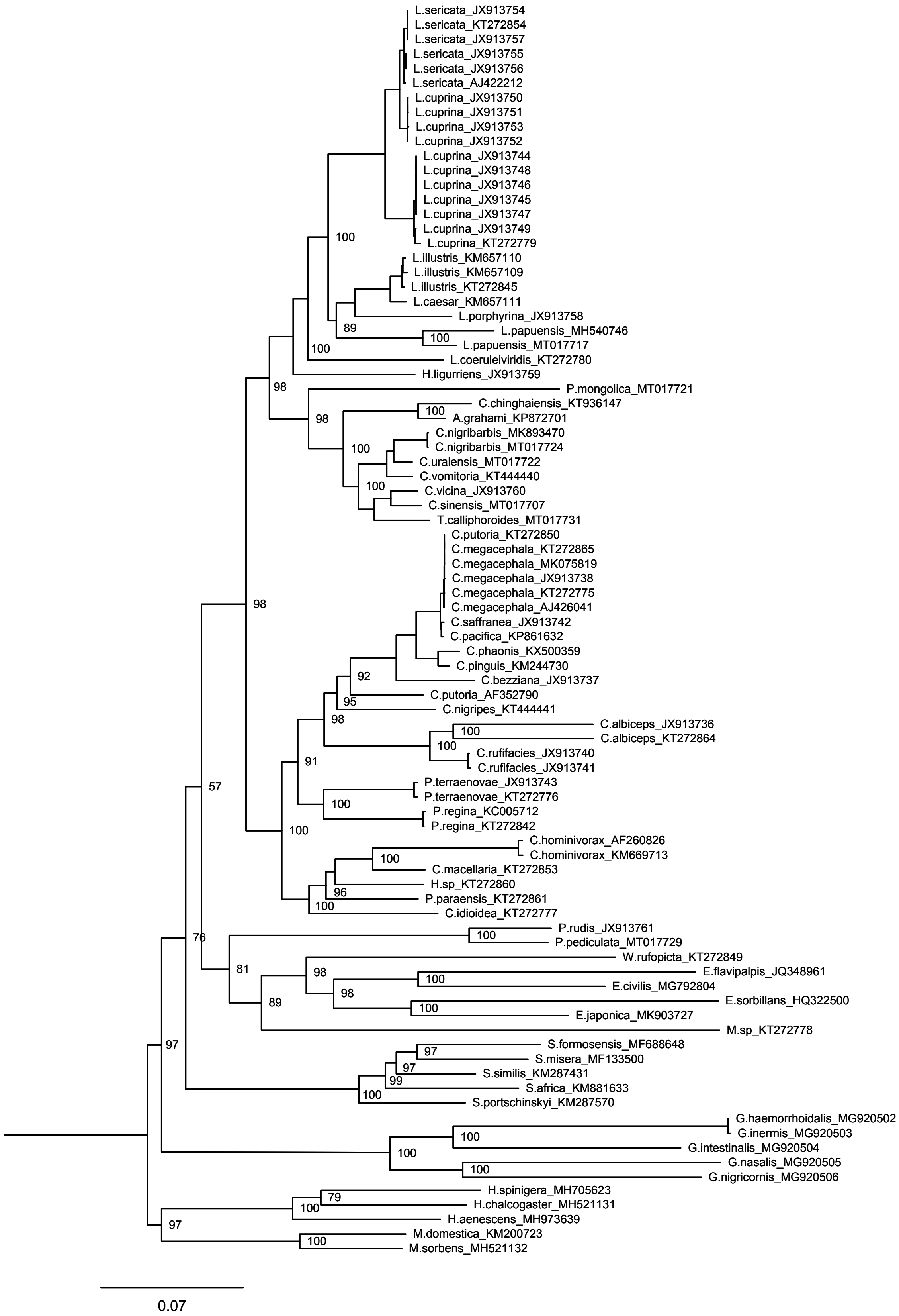

Fig.S7. The Phylogenetic analyses of 34 calliphoridae species (including 63 mitogenomes sequences) within the larger context of the other 18 Oestroidea species were constructed based on the sequences of 13PCGs (excluding the termination codons) and two rRNAs using maximum likelihood (ML) methods. Numbers on branches are bootstrapping replicates (BP).
